# Gait Partitioning Methods: A Systematic Review

**DOI:** 10.3390/s16010066

**Published:** 2016-01-06

**Authors:** Juri Taborri, Eduardo Palermo, Stefano Rossi, Paolo Cappa

**Affiliations:** 1 Department of Mechanical and Aerospace Engineering, Sapienza University of Roma, Via Eudossiana 18, Roma I-00184, Italy; eduardo.palermo@uniroma1.it (E.P.); paolo.cappa@uniroma1.it (P.C.); 2 Department of Economics and Management, Industrial Engineering (DEIM), University of Tuscia, Via del Paradiso 47, Viterbo I-01100, Italy; stefano.rossi@unitus.it; 3 MARLab, Movement Analysis and Robotics Laboratory, Neurorehabilitation Division, IRCCS Children’s Hospital “Bambino Gesù”, Via Torre di Palidoro snc, Fiumicino (RM) I-00050, Italy

**Keywords:** gait phase partitioning, gait pattern, wearable sensors, footswitches, inertial measurements units (IMU), electromyography (EMG), opto-electronic system, force platform

## Abstract

In the last years, gait phase partitioning has come to be a challenging research topic due to its impact on several applications related to gait technologies. A variety of sensors can be used to feed algorithms for gait phase partitioning, mainly classifiable as wearable or non-wearable. Among wearable sensors, footswitches or foot pressure insoles are generally considered as the gold standard; however, to overcome some inherent limitations of the former, inertial measurement units have become popular in recent decades. Valuable results have been achieved also though electromyography, electroneurography, and ultrasonic sensors. Non-wearable sensors, such as opto-electronic systems along with force platforms, remain the most accurate system to perform gait analysis in an indoor environment. In the present paper we identify, select, and categorize the available methodologies for gait phase detection, analyzing advantages and disadvantages of each solution. Finally, we comparatively examine the obtainable gait phase granularities, the usable computational methodologies and the optimal sensor placements on the targeted body segments.

## 1. Introduction

Human gait is a complex and cyclical process requiring the synergy of muscles, bones, and nervous system [1], mainly aimed at supporting the upright position and maintaining balance during static and dynamic conditions [2]. The gait cycle is defined as the period of time from the initial contact of one foot to the following occurrence of the same event with the same foot. Several partitioning models, with different levels of granularity, have been proposed depending on the different clinical aims: the model including two main phases, *i.e.*, stance and swing, is generally the most adopted [3,4,5,6,7] even though a larger number of phases—three [8,9,10,11], four [12,13,14,15,16,17,18], five [19,20,21,22,23,24], six [25,26,27] or more [28,29,30,31,32,33]—must be considered to tackle some particular issues. The correct discrimination of gait phases can be considered the starting point for several scientific applications, such as: (i) the evaluation of gait recovery status in patients after interventions or rehabilitation treatments [34,35,36,37]; (ii) the classification of daily life activities [5,38,39]; (iii) the synergistic control of robotic devices for the recovery of lower limb mobility [18,40,41,42]; (iv) athlete coaching [43,44,45]; and, (v) finally, distinguishing between normal and pathological gait [17,46].

Regardless of the computational methodologies adopted, different sensor systems can be used to capture gait phases. Nowadays, wearable sensors are largely used to perform gait segmentation; foot pressure insoles [6,10,26,47,48] or footswitches [16,20,21,25,49] represent the gold standard in gait segmentation since each gait phase can be associated with a specific value of the sensor output [50]. Alternatively, accelerometers [4,5,19,51,52,53,54,55,56,57,58,59], gyroscopes [7,11,15,17,18,27,38,41,60,61,62], inertial measurement units (IMUs) [3,9,14,34,40,46,63,64,65,66,67], and electromyography (EMG) signals [22,28,33,35] are widely used to feed algorithms for gait phase discrimination. Also the data fusion of above-mentioned sensors has been widely used [12,24,30,32,36,37,68,69,70,71,72,73,74,75]. As concerns indoor environments, non-wearable sensors, such as optoelectronic systems [13,29,76,77,78,79,80] or force platforms [81], are widely adopted. Ultrasonic sensors [82] and electroneurography [83] are proposed marginally in literature. 

The present review is focused on the evaluation of pros and cons for each sensor system, to establish selection criteria for the most suitable solution, based on the specific requirements. Specifically, the literature was reviewed to answer the following three research questions: (i) which is the most appropriate sensor choice depending on the granularity of the gait cycle? (ii) which is the most appropriate choice of body segment for sensor placing? and, (iii) which is the most appropriate computational methodology to be applied depending on the selected sensor?

## 2. Experimental Section 

### 2.1. Search Strategy

Scopus, Google Scholar, and PubMed databases were used to perform a literature search on the topic of gait phase partitioning. The electronic search was conducted in September 2015. Keywords included: *gait events, gait phases*, and their combinations with the words: *partitioning, detection, classification*, *and recognition*. In addition, wildcard symbols, such as hyphens or inverted commas, were used to consider all possible variations of root words. To avoid missing some important studies, a cross referencing was applied from each article found during electronic search. A literature search was performed by Taborri.

### 2.2. Inclusion Criteria

Articles obtained thorough these searches were evaluated using the title and abstract. The articles were included in this systematic review when they met the following criteria: (i) they were written in English; (ii) they were published from January 2000 to September 2015. We excluded conference proceedings when a journal article published by the same authors with the same contents was already included.

### 2.3. Data Extraction

Publications included in this systematic review were downloaded into Mendeley for screening. In order to make the review readable and focused on the authors’ intention, as claimed in the Introduction section, a data extraction was conducted based on major themes: (i) the granularity of the gait cycle ; (ii) sensor placement; and, (iii) method and performance of gait phase classifications. 

### 2.4. Quality Assessment

A quality assessment of the found articles was provided in addition to the systematic review; in particular, publications were subject to seven criteria, as shown in Table 1, in accordance to Campos and colleagues [84]. Independently, Taborri, Palermo and Rossi used the seven criteria to assess the quality of the publications. Any discrepancy among two reports was adjudicated by the third one.

**Table 1:** Criteria for methodological evaluation. “Y” stands for Yes, “N” stands for No, NA stands for “Not-Applicable”.

Criteria	Possible Outcomes
Is the research question well stated?	Y/N
Is the sample/population identified and appropriate?	Y/N
Are the inclusion/exclusion criteria described and appropriate?	Y/N/NA
Is the same data collection method used for all respondents?	Y/N
Are important baseline variables measured, valid and reliable?	Y/N/NA
Is the outcome defined and measurable?	Y/N
Is the statistical analysis appropriate?	Y/N/NA

## 3. Results and Discussion

### 3.1. Search Strategy Yield

The electronic search of previously-mentioned database identified 298 published studies, while 33 further articles were found with cross referencing evaluation. With the application of inclusion/exclusion criteria 72 studies were considered in the present systematic review and the quality assessment protocol identified 32 papers that will be discussed in the following paragraphs.

Since in the examined papers the nomenclature was not coherent, we decided to standardize the name of the gait phases, as synthetized in Table 2 for granularity of gait phases equal to two, three, four, five, six, seven and eight.

**Table 2:** Nomenclature for different granularity of gait phases.

Granularity	Gait Phases																			
Two Phases	Stance														Swing					
Three Phases	First Rocker		Second Rocker												Swing					
Four Phases	Heel Strike				Flat Foot						Heel Off				Swing					
Five Phases	Heel Strike			Flat Foot				Heel Off				Toe Off			Swing					
Six Phases (a)	Initial Contact		Loading Response				Mid Stance			Terminal Stance				Pre Swing	Swing					
Six Phases (b)	Loading Response			Mid Stance				Terminal Stance				Pre Swing			Swing 1			Swing 2		
Seven Phases	Loading Response			Mid Stance				Terminal Stance				Pre Swing			Initial Swing		Mid Swing		Terminal Swing	
Eight Phases	Initial Contact	Loading Response				Mid Stance			Terminal Stance				Pre Swing		Initial Swing		Mid Swing		Terminal Swing	
Gait [%]	0														60			100		

The distribution of the 72 papers based on the utilized sensors and the granularity of the gait is shown in Table 3. The large number of methodologies based on inertial sensors reported in literature can be justified by the significant decrease of cost and increase of popularity in research applications. The inertial quantities, linear accelerations and angular velocities, present typical waveform features during a gait cycle. Thus, it is possible to obtain satisfactory performance in gait phase detection both with threshold-based methods and machine-learning approaches. In addition, these solutions permit the sub-partitioning of the swing phase. Conversely, the swing sub-phases are not recognizable through footswitches or foot pressure insoles, as testified by the lower number of papers. However, these sensors represent the gold standard for stance sub-phase detectiont. As a direct consequence, these sensors are used as a reference system to validate the proposed innovative methods in the majority of the cited papers. The highest percentage was related to the combination of direct measures of the foot contact with the ground and IMU signals. In fact, data fusion allows researchers to obtain high performance in discrimination, reliability of the measures, and a large set of gait phase model with all the possible granularities. EMG-based papers showed a lower diffusion, especially due to the needed heavy post-processing, such as filtering, rectification, *etc.* The relatively high number of paper related to the opto-electronic systems confirms that this approach still represents the most popular one in indoor conditions. In addition, opto-electronic systems remain the most widespread technology installed in clinical laboratories, since they are considered the gold standard for routine gait analysis and no additional sensors are needed for gait phase partitioning. Indeed, inertial sensors are nowadays used only in research applications**.** As regards the use of electroneurogram (ENG) signal and ultrasound sensor to perform the gait partitioning, the lowest diffusion of these methods was essentially due to the more invasive methodology of ENG and to the heavy computational load required by the ultrasonic systems. Finally, we decided to exclude other approaches, such as camera and vision-based methods, since the outcomes were only related to other characteristics of the gait, such as gait activity recognition [85,86,87] and gender recognition [88,89,90].

**Table 3:** Examined papers for each sensor as a function of granularity of the gait phases and relative percentage with respect the total of cited papers. NA stands for “Not-Available”.

Sensors		Gait Phase Granularity								
		#	%	2	3	4	5	6a/6b	7	8
a	Footswitches	5	6.9%	NA	NA	[16]	[20,21]	[25,49]	NA	NA
b	Foot pressure insoles	5	6.9%	[6,47]	[10]	NA	NA	[26,48]	NA	NANA
c	Linear Accelerometers	12	16.7%	[4,5,51,52,53,54,55,56]	NA	[52,57,58]	[19]	[59]	NA	NA
d	Gyroscopes	11	15.3%	[7,27,60,61]	[11,41]	[15,17,18,27,38,60,62]	NA	[27]	NA	NA
e	Inertial Measurement Units	11	15.3%	[3,40,63]	[9]	[14,34,46,64]	[65]	NA	[66]	[67]
f	Combination (a)/(b) with (c)/(d)	14	19.4%	[37,68,69,70,71]	NA	[12,72,73]	[24,74]	[75]	[30,32,36]	NA
g	Electromyography	4	5.6%	NA	NA	NA	[22]	NA	[28,35]	[33]
h	Electroneurography	1	1.4%	[83]	NA	NA	NA	NA	NA	NA
i	Ultrasonic	1	1.4%	NA	NA	[82]	NA	NA	NA	NA
l	Opto-electronic systems	7	9.7%	[76,77,78,79,80]	NA	[13]	NA	NA	[29]	NA
m	Force platforms	1	1.4%	[81]	NA	NA	NA	NA	NA	NA
	Total	72	100%	-	-	-	-	-	-	-

### 3.2. Solutions Based on Wearable Sensors

#### 3.2.1. Footswitches

The gold standard in the field of the gait phase detection is represented by sensors able to directly detect the foot contact with the ground during a gait cycle. From this perspective, footswitches are commonly used for measurements of time gait parameters. These sensors are low-cost, they require simple signal conditioning and post-processing, and they provide high accuracy in gait phase detection. Actually, footswitches are often used to validate algorithms based on other types of sensors [17,18,38]. However, they present several disadvantages: (i) the number of the detectable gait phases is limited, since the sub-phases of the swing cannot be discriminated; (ii) the placement of the sensors on patients with pathological gaits affects the accuracy and reliability [91]; (iii) the wire connections can decrease the system service life [27]; and, (iv) the force generated in the gait cycle cannot be isolated by the concurrent effects induced by the movement of the center of mass [12].

To better clarify the possible granularity achievable with footswitches, three papers with different gait phases were thoroughly investigated. A method to segment the output of three footswitches—placed on heel, first metatarsus and fifth metatarsus—was presented in [16]. The proposed algorithm allowed the detection of four gait phases and it was tested on level walking data gathered from five healthy children and five patients (two hemiplegic children, one with Parkinson’s disease, one with vestibular schwamnonas resection, and one with total hip arthroplasty). The output of the footswitches was digitalized and then fed to the algorithm. The steps implemented in the algorithm can be summarized as follows: (i) initial segmentation of the signal to identify the gait cycles, *i.e.*, the period when the output of the footswitches was equal to zero; (ii) generation of sequence of gait phases based on high and low status of footswitches; (iii) computation of the time length of each estimated cycle; and, (iv) merging the cycles composed of two or three phases and checking that the time length of the merged cycles was less than the mode of the time length, evaluated on the cycles, articulated in four phases. The comparison with a manually performed discrimination of gait phases showed an accuracy of 100% in healthy subjects and 98% in patients. The possibility of detecting atypical cycles was also demonstrated. 

Skelly *et al.* [20] proposed a real-time algorithm for the detection of five gait phases. The algorithm was based on two levels: the first level was a fuzzy logic based on the rule set with nine standard gait cycles and its role was to estimate the above-mentioned five gait phases; the second one was a supervisor to check the duration of the gait cycles previously estimated and to discard them if the time was less than 25% of the mean duration of the standard cycles. The input of the algorithm was the signal gathered by four footswitches placed as follows: two on the heel, one on the first metatarsus and one on the fifth metatarsus. The signal was low-pass filtered at 25 Hz, digitalized with a filter at 1 Hz and smoothed at 7 Hz. The algorithm was tested on three paraplegic adult patients and the errors in the estimation were less than 11% on average for all phases. 

Finally, Bae and colleagues [25] introduced the application of a Hidden Markov Model (HMM) to the outputs of four footswitches, placed on hallux, heel, first metatarsus and fifth metatarsus. This methodology identified six gait phases, which represent the maximum recognizable by footswitches according to the literature. The algorithm was a HMM with six hidden states, trained with a standard gait cycle and it was tested on one healthy adult subject and one patient with Parkinson’s, examined pre- and post-drug treatment. The results showed the possibility of distinguishing normal and pathological gait cycles. 

From an overall examination of the above-mentioned three studies, it emerges that the accuracy of the algorithm for gait phase detection decreases with the increase of the granularity of the gait cycle. Moreover, the footswitches output was suitable to feed both fuzzy inference systems and machine-learning schemes with simple or no signal processing and these can be used on healthy subjects and on patients. 

#### 3.2.2. Foot Pressure Insoles

The advantages and disadvantages of gait partitioning methods based on foot pressure insoles are similar to those related to the footswitches, as they are based on the same principle. Nevertheless, a foot pressure insole could offer better performance with respect to footswitches, as it allows recording the contact of the full foot with the ground, providing a more punctual measure, which does not depend on the placement of the footswitch.

Two papers were here reported in details to show the two computational methodologies: the threshold approach and the machine-learning method.

Catalfamo *et al.* [6] introduced a method based on the activated area in the insole for the discrimination of two phases for gait analysis application. Two algorithms were tested on walking trials of ten healthy adult subjects. The first algorithm, addressed as force-detection, discriminated between the stance phase and the swing phase, imposing a weighted threshold value obtained from the evaluation of maximum and minimum of the force acquired during the entire cycle. The stance phase occurred when the sum of the outputs of all pressure sensors was above the threshold value. The second algorithm, addressed as area-detection, was based on the information of which insole area was loaded. The results obtained from the comparison with force platform output showed better performance in terms of timing delay for area-detection method with respect to the force-detection one.

A methodology for partitioning based on a model including more phases was proposed by Crea *et al.* [26] using two synchronized wireless foot pressure insoles. Each insole was equipped with 64 optoelectronic pressure sensors. The method was validated with 750 steps of walking trials related to five healthy adult subjects and it allowed partitioning the gait cycle into six phases. The algorithm was based on a HMM, trained by means of a leave-one-out cross validation. The output of pressure insoles was post-processed with a Laplace surface smoothing algorithm to eliminate the outliers, and the ground reaction force, the center of pressure and their derivatives were computed; then all the variables were processed by the algorithm. The system performance were evaluated by assuming as a reference the manually performed gait phase discrimination and by determining accuracy and time delay; an accuracy of 95% was observed and there was an average time delay of 3 ms, with maximum of 7 ms, in the transition between Swing 1 and Swing 2. 

From the examined papers, the use of the information associated with the pressed area of the insole rather than the overall exerted force value are more effective in gait partitioning, since they permit a punctual measure of the contact foot/ground. In addition, the use of a machine-learning algorithm and additional computed variables, such as the derivatives of the ground reaction force, appeared to be a more useful and effective approach for the detection of gait phase with a lesser time delay. The second previously examined paper showed as the combination of two pressure insoles discriminated also a sub-phase of the swing period even though the associated computational load was increased. Thus, this approach could present some keys in particular applications like the design and control of bilateral un-tethered wearable exoskeletons.

#### 3.2.3. Linear Accelerometers

Accelerometers tend to be the most used solution for ambulatory gait analysis and different combinations and placements of these type of sensors were described in literature with the aim of recognizing gait phases. Accelerometers, and other inertial units, are miniaturisable, low powered, durable, inexpensive, highly mobile, and readily available sensors [92]. In addition, with respect to solutions based on footswitches or foot pressure insoles, the analysis of the acceleration allowed researchers to recognize a greater granularity of gait cycles, such as the sub-phases of the swing phase. The use of the accelerometers implies some critical issues: (i) the need for gravity compensation in the computation of body segment acceleration; (ii) the extent of computational load required in post-processing; (iii) the presence of drift error in the position data computed by a double numerical integration of the signal; and, (iv) the calibration procedure to correctly place the sensors on the body segment. 

As concerns sensor systems based on linear accelerometers, the first categorization criteria could consist in the accelerometer position on the body; hence four representative papers, differing in targeted body segments and the sensor positioning, were discussed here.

Selles *et al.* [4] showed the feasibility of an algorithm based on linear acceleration measured at the shank for the detection of two gait phases in the exoskeleton control. Linear acceleration of the shank in longitudinal and antero-posterior axis was acquired from level walking of fifteen healthy adult subjects and ten unilateral transtibial adult amputees. The cadence of the gait was set by means of a metronome at different frequencies. Linear acceleration was low-pass filtered at 0.75 Hz and a search window was used to find the local minima in each recorded gait cycle. The first local minimum in the longitudinal acceleration represented the start of the stance, and the last one in the antero-posterior acceleration was the end of the stance phase. The algorithm was trained by means of a subset of the participants (five healthy subjects and two amputees), while the remaining subjects of the two groups were used to test it. The results showed a correlation of 0.99 for healthy subjects and 0.97 for amputees with respect to the reference data gathered by a force platform. 

Rueterbories *et al.* [57] validated a more sophisticated threshold-based algorithm by means of radial and tangential acceleration of the foot. The resultant of the accelerations, the low-pass filtered acceleration at 6 Hz, and the filtered acceleration with moving average technique, were the inputs of the rule-based algorithm. A combination of threshold values of the previously mentioned three variables allowed the detection of four phases. The real-time detection was investigated in the adult population both on ten healthy subjects and on ten subjects with hemiplegia during level walking with a metronome. Using footswitch signals as reference, a True Positive Rate, which represents the correct estimation of gait phases, of 100% in both groups, and a True Negative Rate, which represents the correct estimation of non-transition among phases, of 95% in both groups were found.

A more complex sensor system was introduced by Mijailovic *et al.* [53] for the discrimination of stance and swing phases by using a tri-axial linear accelerometer for each of the three segments (thigh, shank and foot) of each side of the lower limbs. The algorithm was based on a multilayer perceptron trained by walking data of a healthy adult subject and validated by walking data relative to four healthy adult subjects. The study, compared with reference signals obtained by four footswitches, demonstrated the better accuracy obtainable via the linear acceleration of the foot in the sagittal direction. In addition, it was demonstrated that filtered data, with a simple moving average technique, generated better performance in terms of accuracy with respect to raw data (incorrect classifications equal to 8% *vs.* 11%).

A useful method to overcome limitations related to the placement of the sensitive axis of the accelerometers was described by Patterson and Caulfield [59]. The authors used one tri-axial accelerometer placed on the dorsi of the feet to classify seven gait phases. The algorithm was fed by means of the resultant acceleration to overcome the issue of a precise sensor localization on the foot. The time history of the resultant acceleration showed a typical pattern during the gait cycle: (i) a rest period during Flat Foot; (ii) the first oscillation after rest represents the terminal stance; and, (iii) the swing phase presents two humps, the first one is the Pre Swing, while the second one is the Loading Response. The potential of the linear acceleration to detect the sub-phases of the swing was also demonstrated; in fact the Mid Swing was recognized as the first local minimum after the Toe Off. Patterson and Caulfield assessed the possibility of also using this algorithm to differentiate between several walking speeds, testing the algorithm on six healthy adult subjects.

From the discussion of the four examined papers, we can state that the use of a linear accelerometer requires a signal shaping procedure to improve the performance in gait partitioning. Moreover, the implementation of a complex machine-learning algorithm is not mandatory; in fact both longitudinal and antero-posterior linear acceleration show specific peaks at the start and end of the stance phase, easily detectable by means of the application of a threshold algorithm. Thus, linear acceleration was often used to feed algorithms specifically designed to detect two gait phases. From the comparison of different body segments, the sagittal acceleration of the foot was found to be the optimal choice to obtain the best results, regardless the given computational methodology. Moreover, the use of the resultant of the acceleration was a valid suggestion to eliminate the effect of an incorrect sensor placement on the body segment.

#### 3.2.4. Gyroscopes

The use of the angular velocity as a variable to perform gait partitioning reached a greater popularity in recent decades and it was preferred to other inertial variables [52]. Angular velocity is not influenced by the gravity and the waveform is not affected by the vibrations that occurred during the heel strike [93]. Moreover, a careful placement of the sensor on the body segments is not mandatory [94]. As acceleration, angular velocity is affected by drift if the angle needs to be computed.

As for the linear accelerometers, one of the principal issues is related to their placement and seven papers were reported in the following, taking into account this criticality. Catalfamo *et al.* [61] proposed a method based on the angular velocity of the shank for the detection of two phases to be implemented in the control system of an electrical stimulator. The sagittal angular velocity was acquired for seven healthy children during level and incline walking trials. The signal was low-pass filtered at 35 Hz, that represented the optimal cut-off frequency in the tested range of 5–40 Hz, and data from two subjects were used to set the threshold value of the algorithm. The algorithm was based on the detection of two negative peaks in the angular velocity waveforms. The start of the hypothetical stance phase was estimated as the instant when the signal was > 0.2 V for at least 40 ms; then, the first negative peak represented the start of the stance (IC). Successively, in order to discover the actual start of the swing, a waiting window was imposed to avoid the detection of false negative peaks and after 200 ms the first negative peak was considered as the end of the stance (TC), that is the start of the swing. The validation was carried out by the comparison with pressure insole data and the results showed a repeatability of 98% among the subjects. The timing delay was lower than 25 ms for IC in all conditions, with the highest value in incline up walking, and lower than 74 ms for TC in all conditions, with the highest value in incline down walking. The authors also performed a test for the computational load and they demonstrated the feasibility of the algorithm in real-time with a computation speed nine times lower than the algorithm based on wavelet analysis.

Mannini *et al.* in three studies [15,38,95] applied the HMM to the sagittal angular velocity of the foot in order to identify four gait phases. The angular velocity of the foot regularly presents three typical patterns in each gait cycle: (i) two negative polarity humps at Heel Strike and Heel Off; (ii) a plateau in Flat Foot, which follows the first negative hump; and, (iii) a positive polarity hump after Heel Off. This periodical signal was low-pass filtered at 15 Hz and perfectly fed a probabilistic model, such as the HMM [96]. The authors highlighted the greater accuracy of this algorithm with respect to other machine-learning approaches, such as Gaussian Mixture, Support Vector Machine and Linear Discriminant Analysis, and the lower timing delay with respect to a threshold method. In the three studies, several conditions of walking were tested, level and incline at different speeds, as well as the possibility of discriminating between different motor activities, such as walking and running. The methodology appeared to be efficient both on data generated from healthy subjects and patients with pathological gaits. In the last paper, they presented also a simplified version of the Viterbi algorithm, which is the core of the HMM for the estimation of the gait phases, in order to allow the real-time application of the method. Generally, the HMM fed with angular velocity of foot showed a true positive rate and true negative rate of 99% on average. 

Cappa’s research group [17,18,27] in recent years focused on the gait partitioning by means of gyroscope data in order to implement a control system for pediatric exoskeleton. They introduced a novel algorithm based on a hierarchical weighted decision on the output of two or more scalar HMMs. The methodology was tested both on healthy adult subjects, healthy children and children with hemiplegia, in different walking trials, *i.e.* level and incline, at different velocities. The algorithm allowed researchers to estimate the most likely sequence of four gait phases—Heel Strike, Flat Foot, Heel Off and Swing—by means of the sagittal low-pass filtered (30 Hz) angular velocity of foot, shank and thigh. The foot angular velocity appeared to be the most suitable for gait detection, reaching true positive rate and true negative rate of 99% in healthy subjects and more than 85% in children with hemiplegia. The distributed algorithm afforded the optimum compromise among high accuracy, minimum number of sensors and less computational load. Moreover, the authors validated the possibility of avoiding the subject-specific training of the HMM by means of the identification of a standardized parameter set to train the model. These findings raised the possibility of reducing the complexity of the sensor system embedded in the active orthoses. Finally, a comparison among different granularity of gait phase model were tested, and the performance decreased with the increase of granularity.

As a general conclusion related to sensor systems based on gyroscopes, we affirm that angular velocity represents the most suitable variable for the detection of four and six gait phases by means of a machine-learning algorithm since gyroscope output shows periodic and repeatable patterns during the gait cycle. The sagittal angular velocity of the foot was the best candidate to reach better performance, while shank and thigh have to be used together to obtain an accuracy > 90%. Finally, gyroscope outputs also allowed for the discrimination of gait phases during daily activity, *i.e.*, different walking conditions.

#### 3.2.5. Inertial Measurements Units (IMUs)

In IMUs the data fusion of angular velocity and linear acceleration permits to compensate for the drift error. The combination of different inertial quantities permits the evaluation of different typologies of first contact of foot with the ground, which represents an important index for the assessment of healthy status of a subject, by means of the estimation of the foot orientation. Moreover, IMU systems allow researchers to compute spatio-temporal parameters, that are stride length, cadence, *etc.*, other than gait phases.

The different combination of sensors was chosen as representative for the sensor system based on IMU and four papers were reported. The combination of two-axis accelerometers (radial and tangential) and one-axis gyroscope (sagittal) on shank was introduced by Kotiadis *et al.* [9]. The discrimination of a three phase model was carried out by four different algorithms tested on one post stroke subject in different conditions of walking on the ground. Each algorithm started with the identification of the baseline of the used signal and with the set of threshold values for classification. The first algorithm used the radial linear acceleration, the second a combination of radial and tangential acceleration, the third the angular velocity, and the last one the combination of all three inertial variables. All algorithms were compared with the discrimination computed by an opto-electronic system and one footswitch on the heel. The best performance in terms of timing delay was reached by the algorithm based on the three signals (40 ms); however a similar result (50 ms) was obtained by the algorithm applied on the angular velocity. This finding induced researchers to consider the gyroscope as the best solution to reduce the number of sensors.

Lau *et al.* [14] evaluated the performance of a network of sensors obtained from different combinations of linear accelerometers and gyroscopes attached to the thigh, shank and foot. The algorithm was able to determine a three gait phase model. It was found that some specific turning points, which represented the change in the gradient sign of the signal, could effectively identify the required gait events by means of the application of a threshold method. A performance index based on the ROC analysis, in particular on the value of the area under the curve, and the timing variation was used to find the best combination of sensors. The algorithm was tested with three healthy adult subjects and ten patients with hemiplegia and it was shown that the best turning points were: (i) the minimum turning point in acceleration of thigh in transverse plane for First Rocker; (ii) the minimum turning point in angular velocity of shank in frontal plane for Second Rocker; and, (iii) the maximum turning point in acceleration of foot in transverse plane for Swing. 

A hybrid method based on a feed-forward neural network (FNN) embedded in a HMM was introduced by Evans and Arvind [65] for detecting five gait phases. The algorithm, compared with ground reaction force signal, was tested with four walking trials of five healthy adult subjects, which were equipped with seven IMUs, placed on thigh, shank and foot of each side and on the pelvis. FNN was well suited for recognizing patterns in high dimensional sensor input, however the dimension of the dataset had to be fixed. This constraint represented a limit in real-time condition and it was overcome by the introduction of the HMM. The three components of angular velocity and the three components of linear acceleration were used as the input of the algorithm. The training of the FNN was performed by means of a leave-one-out cross validation and the output of FNN represented the emission for the HMM. True positive rate and true negative rate of the classification were equal to 89% and 97%, respectively.

The combination of linear acceleration, angular velocity and magnetic field strength was used [66] to estimate knee and shank angles in the sagittal plane in order to detect seven gait phases. The discrimination was allowed considering also the angular velocity of both feet. The algorithm was tested on five healthy adult subjects, five elderly subjects and two subjects with dementia; all subjects were equipped with four IMUs, placed on right thigh, right shank and both feet respectively. A rule-based method on the instantaneous value of knee angle, shank angle and angular velocity was applied. The algorithm was shown to be useful in monitoring health status since it permitted to recognize different patterns in the sequence of gait phases.

The findings allow researchers to assess that the combination of more inertial variables produce more accurate results. Using all three inertial quantities synchronously, it was also possible to determine kinematic variables useful to construct a supervisor controller to check the correct estimation of the gait phases based on the value of angle joints of lower limbs. Finally, the merger of angular velocity and linear acceleration reduced the time delay during the discrimination from 100 to 10 ms, obtained with only one inertial variable.

#### 3.2.6. Combination of Footswitches or Foot Pressure Insoles and IMUs

Merging IMU and footswitches or foot pressure insoles outputs permitted researchers to construct a robust algorithm for gait detection, overcoming the limits related to each technology, as demonstrated in three thoroughly discussed papers.

Pappas *et al.* [12] designed an automatic algorithm for gait phase detection based on the simultaneous processing of three footswitches and one gyroscope output. The footswitches were placed on the heel, outer and inner midfoot, while the gyroscope was placed on the heel with sensitive axis in the sagittal direction. The algorithm classified four gait phases and it was based on the on/off status of footswitches and on the angle value derived from the angular velocity. The computation of the angle from the value of angular velocity caused a drift bias error, for this reason a resetting method, based on the zero value during stance phase, was used to remove the integration drift. Before the integration, the angular velocity was band-pass filtered at 0.25–25 Hz; while footswitches output was low-pass filtered at 100 Hz. The validation was conducted by ten healthy adult subjects and six subjects with pathological gait in different inclined walking conditions. The time delay in gait phase identification, obtained in comparison with opto-electronic system results, was less than 70 ms for all phases and it was found a repeatability of the algorithm in the speed range of 0.5–13 km/h. The accuracy in the gait phase detection was 100% for normal gait and 99% for pathological gait.

A fuzzy inference system for detection of seven gait phases was validated in [32]. The aim of this study was the design of a wearable smart device for gait event detection useful for clinical gait analysis. The device was equipped with four footswitches, placed on hallux, first metatarsus, fifth metatarsus and heel, and two IMUs placed on shank and thigh. The inputs of the fuzzy system were the on/off status of the footswitches, which had characteristic patterns during sub-phases of the stance, and the knee angle evaluated by means of the two IMUs, which had characteristic features during the sub-phases of swing. The fuzzy system was constructed with rules based on the high and low activation of the footswitches estimated by a sigmoid function and on the high or low peak in the knee angle. The algorithm was tested with walking trials of six healthy adult subjects. Subjects were asked to perform normal walking trials and trials in which they had to simulate toe-drag and toe-walking patterns. Errors in time delay detection were on average < 70 ms. The limit of the proposed algorithm is related to the detection of Initial Swing and Mid Swing in subjects affected by toe-drag since the knee angle was always lower than the chosen threshold.

Gorsic *et al.* [73] proposed an on-line phase detection using a wearable sensor to control robotic prosthesis. The sensor system consisted in seven multi-component inertial measurement units, placed on thigh, shank, and foot of both lower limbs, and on pelvis, and two pressure insoles. The algorithm was a rule-based method according threshold values set on eight variables: ground reaction force and center of pressure of left and right foot, difference between ground reaction force of the two sides, angular velocity of right and left foot, and sum of knee and hip angles. The rules were set on data of walking trials of five healthy adult subjects. The algorithm permitted researchers to classify four gait phases and it was tested on three elderly amputees. The accuracy in the detection was on average equal to 97%, which was comparable to the value obtained by adopting a HMM approach.

From the reported details of the examined papers, it emerged that the combination of the inertial variables and footswitches or foot pressure insoles implies the measurement of the angular velocity. The data was fused: (i) to increase the number of gait phases by individuating also the sub-phases of the swing, which are Initial, Mid and Terminal; and (ii) to use the angular velocity to compute knee angles. Actually, the knee angle time history is characterized by humps in the Stance phase and in the Swing sub-phases, after which it can be effectively used to further check the footswitch outputs. It is worth noting that for the computation of the knee angle the proximal and distal body segments have to be sensorized with multi-component IMUs.

#### 3.2.7. Electromyography (EMG)

The EMG signal was less popular than other wearable sensor systems due to the inherent higher complexity in acquisition and post-processing. Nevertheless, EMG signal is useful for gait phase detection since lower extremity muscle activity occurs in a repeatable way during gait cycle [97]. The selection of the specific muscles to be monitored is discussed below. 

In two studies Lauer *et al.* [22,28] proposed an adaptive neuro-fuzzy inference system (ANFIS) with a supervisory control system (SCS) to develop a control algorithm for the application in functional electrical stimulation. The algorithm classified five [22] or seven [28] gait phases and it was tested on level walking data gathered on both healthy adult subjects [22] and patients with cerebral palsy [28]. The EMG surface electrodes were placed on *vastus lateralis* and *rectus femoris* and the signals were processed as follows: (i) bandwidth filtering in the range of 0–2 kHz; (ii) full wave rectification; (iii) low-pass filtering at 3 Hz to extract the envelope; and, (iv) computation of the derivate between two consecutive time points in order to quantify the activation status. EMG envelope and the derivative of the signal were used as the input of the detection algorithm, which predicted the gait phase based on subject-specific fuzzy rules according to a Sugeno fuzzy model [98]. The output of the fuzzy system was then fed to a supervisor control to exclude unrelated gait cycles. The validation of the classification outputs, in terms of timing of detection and accuracy, was performed by using an opto-electronic system as a reference. Timing error was on average 130 ms and accuracy on average 90% for both cohorts of subjects. 

Joshi and colleagues [33] proposed a control system for a foot-knee exoskeleton based on the processing of eight EMG outputs, four for each leg, placed on quadriceps, hamstring, *tibialis anterior* and *gastrocnemius*. Four time domain features—mean absolute value, waveform length, variance and slope sign change—and 4th order auto-regressive model were computed for each EMG signal and used to feed the Bayesian Information Criteria (BIC). Successively, the application of a Linear Discriminant Analysis (LDA) allowed researchers to identify eight gait phases. The algorithm was tested with data acquired by one healthy adult subject, which performed ten gait cycles of level walking. The first nine cycles were used to train the classifier and the last one to test it, according with a leave-one-out cross validation. The results were compared with the gait phases detected by means of the sagittal angle of hip joint obtained by an opto-electronic system and partitioned in the eight gait phases by an expert operator. The accuracy of the control algorithm increased on average from 50% to 80% with the combination of the BIC and LDA stage. The main critical issue was related to the observed low repeatability in the construction of the training dataset: a difference of 30% in the accuracy value was found changing the gait cycles tested.

From the overall comparison of the above examined three studies, it emerged that an approach based on threshold rules applied on EMG signals performed better than machine-learning algorithms. This finding can be justified considering the low repeatability of the collected signals during different trials and the consequent difficulty in training the algorithm. However, EMG systems permit the identification of up to eight phases, which is the highest possible granularity in gait partitioning according to the literature.

#### 3.2.8. Electroneurogram (ENG)

The cutaneous afferent activities show predictable and repeatable patterns during the stance phase and a silent period during swing phase [99]. Chu *et al.* [83] presented a novel system for functional electrical stimulation to correct the drop foot in patients with neurological diseases. The cutaneous afferent activities of rats’ sciatic nerve during level walking on a treadmill was recorded to validate the algorithm. The ENG recordings were rectified and bin-integrated in a 5 ms window. Then, the signal was firstly examined by means of a wavelet packet transform to extract characteristic features and secondly, used to fed a Gaussian Mixture Model (GMM) to discriminate stance and swing phase. The GMM was trained by a 30-fold cross validation and tested with the last one trial. The results were compared with the angle joint of the ankle acquired with an opto-electronic system. The findings showed an accuracy of about 95% in all tested velocities and demonstrated the greater potential of the GMM. Further, an excessive detection time delay was observed in the toe-off detection, *i.e.*, start of the swing phase, during trials performed at low speed. The limitation of this study, and in general of ENG, relates to the invasive method and the possibility of detecting only two phases: stance and swing. Moreover, no tests on humans were performed to the authors’ knowledge.

### 3.3. Non-Wearable Sensors

#### 3.3.1. Opto-Electronic System

Kinematic data determined via an opto-electonic system represent the gold standard in gait analysis in indoor environments, as well as for the detection of gait phases and they do not require additional measuring chains. In fact, opto-electronic systems cannot be used outside the laboratory in real life situations. Different kinematic variables can be chosen for the implementation of the algorithm for gait phases detection. Three approaches were thoroughly described in the following.

MacDonald *et al.* [29] discussed the feasibility of a fuzzy inference system for classifying human gait phases using sagittal angles of lower limb joints. This method allowed the recognition of seven gait phases. Data from six healthy adult subjects were collected during twenty trials of level walking on a force platform. The force platform signal of three subjects was used to extract for each trial one gait cycle, which was successively used to set the rules of the fuzzy system. The fuzzy system is based on a Mamdami fuzzy if-then inference mechanism [100] and rules were set based on the amplitude of hip, knee and ankle angles evaluated in the same time frame. The system accurately estimated the seven gait phases, in comparison with the gait phase detection performed by an expert operator, with an average error equal to 2% in the computation of stride length.

A different algorithm for the detection of two phases based on the foot velocity computed from marker trajectory, was proposed by O’Connor *et al.* [80]. The method was compared in terms of timing detection with the use of a force platform on data from fifty-five healthy children and three children with spastic diplegia. More specifically, the protocol consisted in performing three level walking trials at the preferred speed. The heel and toe markers were used to feed the novel algorithm, addressed as Foot Velocity Algorithm (FVA). The vertical component of the markers was low-pass filtered at 7 Hz and a new signal was generated by computing the mean point of the heel and toe marker position at each frame. The proposed algorithm was based on the vertical component of the velocity because it showed typical patterns in gait cycle. The end of the stance phase occurred when the vertical component reached the maximum, while the start of the stance phase was identified as the minimum value. Since several local minima were detected in the vertical velocity, a threshold value on the height of heel marker (< 35% of maximum height) was imposed to properly detect the start of the stance phase. Both in healthy children and in children with diplegia the timing delay in the detection was < 16 ms and < 9 ms for the start and the end of the stance phase, respectively.

A neural network algorithm was presented by Miller *et al.* [77] to detect the stance and the swing phase. Trajectories of heel and toe markers were acquired from 90 healthy young adult subjects, divided in two groups: 49 performed barefoot walking and 41 performed walking in shod/braced condition. The novel algorithm was based on a neural network fed with the heel and toe marker trajectories evaluated in the sagittal plane. The marker trajectories were differentiated using five point numerical differentiation and then low-pass filtered at 20 Hz. The neural network was fed by time series of nine variables: sagittal position, velocity and acceleration of the two markers, foot-floor angle, angular velocity, and angular acceleration. Principal component analysis was used to reduce the number of independent variables. The classifier was trained with data from 50 subjects (29 barefoot and 21 braced) and validated with the remaining 40. The validity of this method was assessed using as a reference the ground reaction force and showed a timing delay of 25 ms for the start of the stance and 40 ms for the end of the stance in braced condition, while 8 ms for both events in barefoot. These findings showed that this algorithm has good performance with several normal and pathological gaits if previously trained. 

Generally, a visual marker based methodology allows researchers to recognize two phases and it could fail in case a higher granularity is required. Alternatively to reach a granularity of seven additional variables have to be computed, e.g., marker velocity in vertical direction and joint angles at hip, knee and ankle level, requiring a greater computational load of the algorithm.

#### 3.3.2. Force Platform

The force platform is a well-suited sensor for easily detecting gait events such as the start and the end of the stance phase by imposing a threshold value to detect when the foot contacts the ground. Nevertheless, in clinical gait analysis, to perform a kinetic evaluation several trials have to be conducted since subjects have to place the foot, without adaptation of the stride, on the multicomponent load cells in their entirety. Moreover, in level walking trials the number of installed force platform, in general one or two, limits the number of consecutive available gait cycles; such a limitation can be overcome in the trials conducted with a treadmill equipped with an embedded single large force platform. 

Roedrink *et al.* [81] proposed a novel algorithm for the on-line detection of stance and swing phases from kinetic data gathered by a single large force platform embedded in a treadmill. The algorithm was tested on data from twelve healthy adult subjects, which performed five trials of level walking. The force data were firstly low-pass filtered at 100 Hz and then converted to center-of-pressure data, which presented a typical ‘butterfly’ diagram characteristic in different gait events. A threshold rule was then applied to discriminate the start and the end of the stance phase of the right and left side. Results were compared to off-line kinematic data gathered by an opto-electronic system. The use of the ‘butterfly’ diagram allowed researchers to obtain good results in terms of timing delay for start of the stance (2.6 ms on average) and discrete results for the end of the stance (31 ms). The combination of this approach with a threshold classifier applied to the discovery of local minima in the time series of total force permitted a decrease of the time delay in the identification of the end of the stance (timing delay 15 ms). The limit of this study is related to the difficulty in reaching satisfactory results in normal gait with a large set of velocities and, in pathological gait, a variation of patterns in the butterfly diagram can be expected. 

#### 3.3.3. Ultrasonic Sensor

Qi *et al.* [82] proposed the development of a low-cost portable tracking system for wireless gait analysis based on an ultrasonic sensor network. An ultrasonic system permitted the recording of the foot motion; in particular the foot displacement was obtained from the movement of the ultrasonic sensor placed on the heel of the subject. The algorithm was based on a spherical positioning technique, which found the intersection area of circles centred at each anchor with radius equal to the measured distance from the transmitter to each anchor. After the computation of foot displacement in frontal and transverse planes, a threshold algorithm based on maximum and minimum displacement in the two directions was applied in order to detect four gait phases. The novel algorithm was tested on ten healthy adult subjects and two adult patients with ankle sprain. The validation was performed via an opto-electronic system and a correlation of 0.97 between the two gait detection systems was observed. The error expressed in terms of timing delay in the identification of the transition was < 90 ms in all four phases. The proposed system had two major issues: (i) the presence of obstacles between transmitter and receiver; and, (ii) the range limited to 20 m between transmitter and receiver. 

### 3.4. General Discussion 

In Table 4 we reported a comparative examination of the previous cited papers by comparing different typical requirements of gait partitioning methods.

1. Which is the most appropriate sensor system based on the required granularity?
  The choice of the number of phases is driven by the application and, in general, a granularity increase is required to discriminate daily activities or for the assessment of pathological gait. In fact, the sub-phases of stance and swing and their duration represent effective indices in the evaluation of pathology severity.
  A granularity of two can be considered sufficient in functional electrical stimulators and in synchronizing the activation of motors in wearable exoskeletons. If the granularity is lower or equal to 6, the footswitches or foot pressure insoles are the most suitable choice due to the best guaranteed accuracy and the easiest required post-processing. Nevertheless, it is recommended to avoid their use in daily application due to their short service life. If the granularity required is higher than 6, the inertial sensors are appropriate in place of the footswitches; in particular it was demonstrated that: (i) angular velocity of foot produces a better performance among other inertial quantities; (ii) only one gyroscope is sufficient to correctly discriminate gait phases in both healthy and pathological gait; and, (iii) if an accuracy close to 100% is required, a trade-off between higher accuracy and number of sensors has to be reached. As regards a granularity of 8, that is the maximum number of sub-phases of the gait cycle according to the literature, the only viable system is based on EMG signals, even though an accuracy not greater than 80% can be obtained.
  Moreover, ENG signal can be used only in the discrimination of two phases; while two, three or four phases can be recognized by ultrasonic sensors.
  As concerns non-wearable sensors, force platforms joined with opto-electronic system perform an accurate measure with the combination of marker trajectories and ground reaction force signals, allowing the application in indoor environment and ambulatory gait analysis.
2. Which is the most appropriate body segment to be sensorized?
  The specific application often imposes the sensor positioning on the targeted body segment, for example, in the design of the exoskeletons. The use of the footswitches requires at least one sensor placed on the heel and it can be the only one if it is sufficient to discriminate two phases. When the heel contact has to be recognized with the maximum achievable accuracy, the use of two footswitches is advisable. To increase the granularity, a greater number of footswitches have to be considered and candidate positions are toe, first and fifth metatarsus. As regard accelerometers and gyroscopes, their recommended position is on the foot with the sensitive axis aligned with the sagittal axis. In case EMG signals are chosen, the Rectus Femoris appears to be the muscle that guarantees the best performance in terms of accuracy and time delay. Finally, in visual based methods, heel and toe markers are sufficient to record all variables useful for discrimination, such as marker trajectories and velocity.
3. Which is the most appropriate computational methodology given the selected sensor?
  The computation methodology has to take into account the time history of the chosen variables. A waveform that shows specific and standard values in correspondence of transition between two phases should be treated with algorithms based on the threshold method or fuzzy inference system with rules set on specific temporal values. Instead, quantities characterized by periodic and repeatable patterns during gait phases, such as angular velocity, linear acceleration, marker trajectories, EMG and ENG signals, should be used to feed machine-learning algorithms, and among these schemes the Hidden Markov Model has demonstrated its superior performance. It is worth noting that the use of EMG to feed machine-learning algorithms requires particular attention in the training stage due to the low repeatability of the signal among several trials.
  Moreover, the previously indicated variables, such as angular velocity, linear acceleration, and EMG, require a specific treatment of post-processing: (i) angular velocity has to be low-pass filtered in the range 15–30 Hz, (ii) linear accelerometer has to be low-pass filtered in the range 1–20 Hz, and (iii) EMG has to be rectified, pass-band filtered in the range 0–2 kHz and the envelope with a low pass filter in the range 3–5 Hz has to be extracted.

**Table 4:** Comparative examination of different gait phase partitioning system based on several requirements. Y stands for “Yes”.

Sensor Systems	Wearability	Low Cost	High Service Life	Critical Sensor Placement	Outdoor Applications	Heavy Signal Post- Processing	All Possible Granularities
Footswitches	Y	Y	-	-	Y	-	-
Pressure insoles	Y	-	-	-	Y	-	-
Accelerometers	Y	Y	Y	Y	Y	-	Y
Gyroscopes	Y	Y	Y	Y	Y	-	Y
IMUs	Y	Y	Y	Y	Y	-	Y
Electromyography	Y	-	Y	Y	Y	Y	Y
Electroneurography	Y	-	Y	Y	Y	Y	-
Ultrasonic	-	-	Y	Y	-	Y	-
Opto-electronic	-	-	Y	Y	-	-	Y
Force platforms	-	-	Y	-	-	-	-

## 4. Conclusions

In recent years, several studies have focused on which sensors have to be used to discriminate gait phases. Classification of gait phases is a fundamental issue in several research fields and, based on the specific application, a selection of the proper sensor and post-processing procedure needs to be carried out. The gold standard, often used as a reference signal for the validation of other methodologies, is represented by direct measurements of the contact between foot and ground by footswitches, foot pressure insoles or force platforms. Nevertheless, indirect measurements by means of inertial systems, electromyography or opto-electronic system could be useful when the discrimination of the swing sub-phases is required and long-lasting measurements have to be conducted. To properly identify the optimal solution depending on the desired accuracy and time delay, the following constrain conditions need to be fully exploited: gait cycle granularity, sensor placements, and computational methodology. 
